# Genetic diversity of *Echinococcus multilocularis* and *Echinococcus granulosus sensu lato* in Kyrgyzstan: The A2 haplotype of *E*. *multilocularis* is the predominant variant infecting humans

**DOI:** 10.1371/journal.pntd.0008242

**Published:** 2020-05-13

**Authors:** Cristian A. Alvarez Rojas, Philipp A. Kronenberg, Sezdbek Aitbaev, Rakhatbek A. Omorov, Kubanychbek K. Abdykerimov, Giulia Paternoster, Beat Müllhaupt, Paul Torgerson, Peter Deplazes

**Affiliations:** 1 Institute of Parasitology, Vetsuisse and Medical Faculty, University of Zürich, Zürich, Switzerland; 2 City Clinical Hospital #1, Surgical Department, Faculty of Surgery of the Kyrgyz State Medical Academy, Bishkek, Kyrgyzstan; 3 Section of Epidemiology, Vetsuisse Faculty, University of Zürich, Zürich, Switzerland; 4 Clinics of Hepatology and Gastroenterology, University Hospital of Zürich, Zürich, Switzerland; Istituto Superiore Di Sanita, ITALY

## Abstract

Alveolar and cystic echinococcosis (AE, CE) caused by *E*. *multilocularis* and *E*. *granulosus s*.*l*., respectively, are considered emerging zoonotic diseases in Kyrgyzstan with some of the world highest regional incidences. Little is known regarding the molecular variability of both species in Kyrgyzstan. In this study we provide molecular data from a total of 72 parasite isolates derived from humans (52 AE and 20 CE patients) and 43 samples from dogs (23 infected with *E*. *multilocularis* and 20 with *E*. *granulosus s*.*l*.).Genetic variability in *E*. *multilocularis* was studied using the concatenated complete sequences of the *cob*, *nad2* and *cox1* mitochondrial genes adding a total of 3,558bp per isolate. The *cob*/*nad2*/*cox1* A2 haplotype was identified in 63.4% of the human and in 65.2% of the dog samples. This haplotype was originally described in samples from Kazakhstan and St. Lawrence Island (Alaska, USA). We also describe here 16 non-previously defined variants of *E*. *multilocularis* (called A11-A26). All haplotypes cluster together within the Asian group in the haplotype network. Based on Fst values, low level of genetic differentiation was found between the populations of *E*. *multilocularis* isolated from different regions within the country. However, high degree of differentiation was found when all the concatenated sequences from Kyrgyzstan are considered as a single population and compared with the population of the parasite from the neighbouring country China. In the case of *E*. *granulosus s*.*l*. the analysis was based in 1,609bp of the *cox1* gene. One isolate from a dog was identified as *E*. *equinus*, while all the other sequences were identified belonging to *E*. *granulosus s*.*s*. In total, 24 *cox1* haplotypes of *E*. *granulosus s*.*s*. were identified including the already described variants: Eg01 (in 6 samples), Eg33 (in 4 samples), EgCl04 (in 2 samples), Eg03 (in 1 sample) and Eg32 (in 1 sample). From the twenty-five other isolates of *E*. *granulosus s*.*s*. a total of 19 non-previously described *cox1* haplotypes were identified and named as EgKyr1 to EgKyr19. The most common haplotype infecting human is the EgKyr1 which was found in 5 isolates.The *cob*/*nad2*/*cox1* A2 haplotype of *E*. *multilocularis* is responsible for the majority of human infections in Kyrgyzstan and is also found in the majority of dogs included in this study. Further similar studies in different parts of Asia could elucidate if it is also the most common variant infecting humans in other countries. It remains unknown if this particular haplotype presents differences in virulence which could have contributed to the emergency of alveolar echinococcosis in Kyrgyzstan. In the case of *E*. *granulosus s*.*s*. it seems that there is no dominant haplotype infecting humans in Kyrgzstan. Further characterization of biological or antigenic features of dominant mitochondrial haplotypes could help to elucidate if they present differences which could be relevant in the diagnostic, pathogenicity or in the host/parasite interaction when infecting humans.

## Author summary

Analysis of the genetic variability in *Echinococcus* species from different endemic countries have contributed to the knowledge in the taxonomy and phylogeography of these parasites. The most important species of this genus, *Echinococcus granulosus sensu lato* and *Echinococcus multilocularis*, co-exist in Kyrgyzstan causing serious public health issues. *E*. *granulosus s*.*l*. causes cystic echinococcosis and *E*. *multilocularis* is the causative agent of alveolar echinococcosis. The most relevant finding of our study is the identification of the *cob*/*nad2*/*cox1* A2 haplotype of *E*. *multilocularis* as the most commonly found in humans and dogs. However, it remains unknown if this variant of *E*. *multilocularis*, based on genetic differences in mitochondrial genes, presents differences in virulence which could have contributed to the emergence of alveolar echinococcosis in Kyrgyzstan. The results also show a number of non-previously described genetic variants of *E*. *multilocularis* and *E*. *granulosus s*.*s*.

## Introduction

Alveolar (AE) and cystic echinococcosis (CE), caused by *Echinococcus multilocularis* and *Echinococcus granulosus sensu lato* respectively, are serious zoonotic parasitic diseases with a considerable socioeconomic impact [1]. While CE has a worldwide distribution, AE is confined to the northern hemisphere; thus, in some countries, both parasites co-exist as is the case of Kyrgyzstan [2]. AE has become an increasing public health problem in Kyrgyzstan, as in other Asian countries [1,3,4,5], with a rise in the hospital incidence from 0–2 cases/year in the early 1990s to 148 cases reported in 2013 (2.6 cases per 10^5^ inhabitants/year) with some districts in Kyrgyzstan reporting up to 58 cases per 10^5^ inhabitants/year [6,7]. A recent detailed account of surgical incidence for AE shows values as high as 11.77/10^5^ in Naryn and 7.94/10^5^ in Osh [8]. CE is also highly endemic in Kyrgyzstan with a hospital incidence which has increased almost three times between 1991 and 2013 (from 5.4 to 15.8 cases/10^5^ inhabitants/year) [6,9]. However, the true incidence of both diseases is likely to have been underestimated as in other places in the world mostly due to underreporting of the diseases [6,10]. The increase of incidence of CE cases has been linked to changes in farming practices and the closing of meat-processing plants after the dissolution of the Soviet Union [4]. In some areas, such as the Naryn region in central Kyrgyzstan, CE prevalence in sheep reaches 64% [11]. The increase in dog population (especially stray dogs) [12] has influenced the rise in the incidence of both diseases. Particularly, in the case of AE high levels of infection in dogs have been reported, for example up to 18% in the Naryn district [13]. On the other hand, the prevalence in red foxes (natural definitive host for *E*. *multilocularis*) can be as high as 64% in the same district [14]. A common factor for the recent increase of the incidences of both diseases is the intensification in poverty after the economic changes since 1991 [4]. There is a sparsity of information on the molecular variability of *E*. *multilocularis* in Kyrgyzstan, with a single study analyzing the EmsB microsatellite in two isolates from a vole and a dog from the Alay valley (Osh region) [15]. In the case of *E*. *granulosus s*.*l*., the only genetic characterization of the parasite in Kyrgyzstan described the presence of *E*. *granulosus s*.*s*., *Echinococcus equinus* and *E*c*hinococcus intermedius* G6/7 in dogs [13]. No data from humans has been reported for *Echinococcus* species. In this study, we aim to investigate the genetic variability of *E*. *multilocularis* and *E*. *granulosus s*.*l*. in Kyrgyzstan. For this purpose, we have an unprecedentedly large number of human samples for both diseases and also dog faeces positives for either *E*. *multilocularis* or *E*. *granulosus s*.*l*.

## Methods

### Ethics approval for use of human samples

The Ministry of Health of the Kyrgyz Republic provided ethical approval for this study and patients signed a consent form to participate in the study.

### Human samples

Sixty-one samples consisting of parasite tissue derived from human patients diagnosed with AE (collected between Sept 2017 and Feb 2019) and twenty-three similar samples isolated from CE patients (collected in January and February 2019) were collected after surgery at the City Clinical Hospital in Bishkek, Kyrgyzstan. The discrimination between AE and CE was made by the surgeons in Kyrgyzstan supported by clinical data together with ultrasound and CT scan, Casoni skin test and histology of the resected liver lesions. For AE patients, the mean age of 26 men patients was 34 years, while the mean age of 35 female patients was 39 years. All patients had primary lesions in the liver, with 62% being located in the right liver lobe, 33% in the left liver lobe and 5% in both lobes. Of all 23 CE patients, 11 were male and 12 female (mean age: 33 and 29 years respectively). The diagnosis was mainly based on abdominal ultrasound scans, therefore there is only one case with an infected lung. All the rest of CE patients had lesions in the liver, with cyst localisation of 70% in the right, and 30% in the left liver lobe. Surgical specimens were stored in ethanol 70% and shipped to the Institute of Parasitology in Zürich, Switzerland for molecular analysis. Genomic DNA was isolated, after washing the samples three times with PBS1X, using the tissue protocol for the DNeasy Blood & Tissue Kit (Qiagen). DNA was used for routine PCR which can differentiate *E*. *multilocularis* from *E*. *granulosus s*.*l*. [16].

### Dog faecal samples

Dog faecal samples from a parallel project studying the prevalence of *Echinococcus* species in Kyrgyzstan were selected for this study. These samples were collected from the ground in 10 villages located in the Alay and Kochkor districts of Kyrgyzstan (Osh and Naryn regions, respectively), during three expeditions carried out in September 2017, February and June 2018. Dog faeces were identified based on morphological criteria and color. In summary, the detection of taeniid eggs in faecal samples was accomplished through a combination of flotation with zinc chloride (density 1.45) and sieving through nylon meshes of 40 and 21 μm size as described before [17]. The sediment retained in the 21 μm mesh was deposited in a flat-sided tube and examined for the presence of taeniid eggs using an inverted microscope. Positive samples to taeniid eggs were selected and centrifuged at 1,000g for 10 min. DNA was isolated from the sediment as previously described [18] and used as a template in a multiplex PCR to discriminate between *E*. *granulosus s*.*l*., *E*. *multilocularis* and other cestodes [16]. In total, 28 samples positive to *E*. *multilocularis* and 24 positives to *E*. *granulosus s*.*l*. were identified and selected for further molecular analysis.

## Molecular analysis

### *E*. *multilocularis*

DNA identified as *E*. *multilocularis* from parasite tissue derived from human samples and from dog faeces was used as a template for the amplification of the full length of three mitochondrial genes. These genes were cytochrome b (*cob*), cytochrome c oxidase subunit 1 (*cox1*) and NADH dehydrogenase subunit 2 (*nad2*) using primers previously described [19]. PCR products were visualized in a 2% agarose gel and purified using the MinElute PCR purification kit (Qiagen) for subsequent Sanger sequencing with the same primers used for amplification. In the case of the *cox1* gene, an extra internal reverse sequencing primer was designed (5’-AGCCACCACAAATCAAGTATCG-3’) (Microsynth, Switzerland). Only electropherograms with clear single peaks were accepted, particular attention was given to samples from dogs taking into consideration that mixed haplotype infections can occur [20]. Electropherograms with double peaks in any of the genes analysed from dog samples were considered to represent infections with two or more haplotypes, and therefore, were not considered in the study. Sequencing analysis was performed with Geneious v11.1.5 and the sequence of each gene was assembled following the reference mitochondrial genome for *E*. *multilocularis* (Accession number AB018440). Concatenated sequences of the *cob* (1,068bp), *nad2* (882bp) and *cox1* (1,608bp) genes for each DNA sample (adding 3,558bp) were aligned together with other similar available sequences from representative and distinct haplotypes from Asia (A1-A10), Europe (haplotypes E1-E5) and North America (N1-N2) from investigations by Nakao et al. [19]. Alignments were exported as a PHYLIP and NEXUS extensions and used as input for TCS v1.21 [21] and PopArt [22] for the identification of haplotypes, network construction and estimation of diversity indexes. Genetic distance between two subpopulations was analyzed by pairwise fixation index (Fst) calculated and statistically compared using Arlequin 3.5 [23].

### *E*. *granulosus s*.*l*.

DNA identified as *E*. *granulosus s*.*l*., from parasitic tissue derived from humans cysts and dog faeces, was used for amplification of the *cox1* gene using primers previously described [24]. Sequences were assembled using the *cox1* haplotype Eg01 as reference (JQ250806), with the same tools as explained above for *E*. *multilocularis*. As in the case of *E*. *multilocularis*, only electropherograms with single peaks were included in this study. Firstly, the genotypes of *E*. *granulosus* (G-system) present in the isolates characterized in this study were identified based on 366bp of the *cox1* gene. The original reference sequences for the G1, G3 and other genotypes of *E*. *granulosus s*.*l*. described by Bowles et al [25] were used as reference in alignments for comparison with the sequences acquired in the present study. Subsequently, for the identification of the *cox1* haplotypes of *E*. *granulosus s*.*s*. we included all sequences of the same length (1,609bp) deposited in GenBank. Network construction, estimation of diversity indexes and pairwise fixation index (Fst) were acquired as explained above for *E*. *multilocularis*.

## Results

### *E*. *multilocularis*

**Fig 1:**
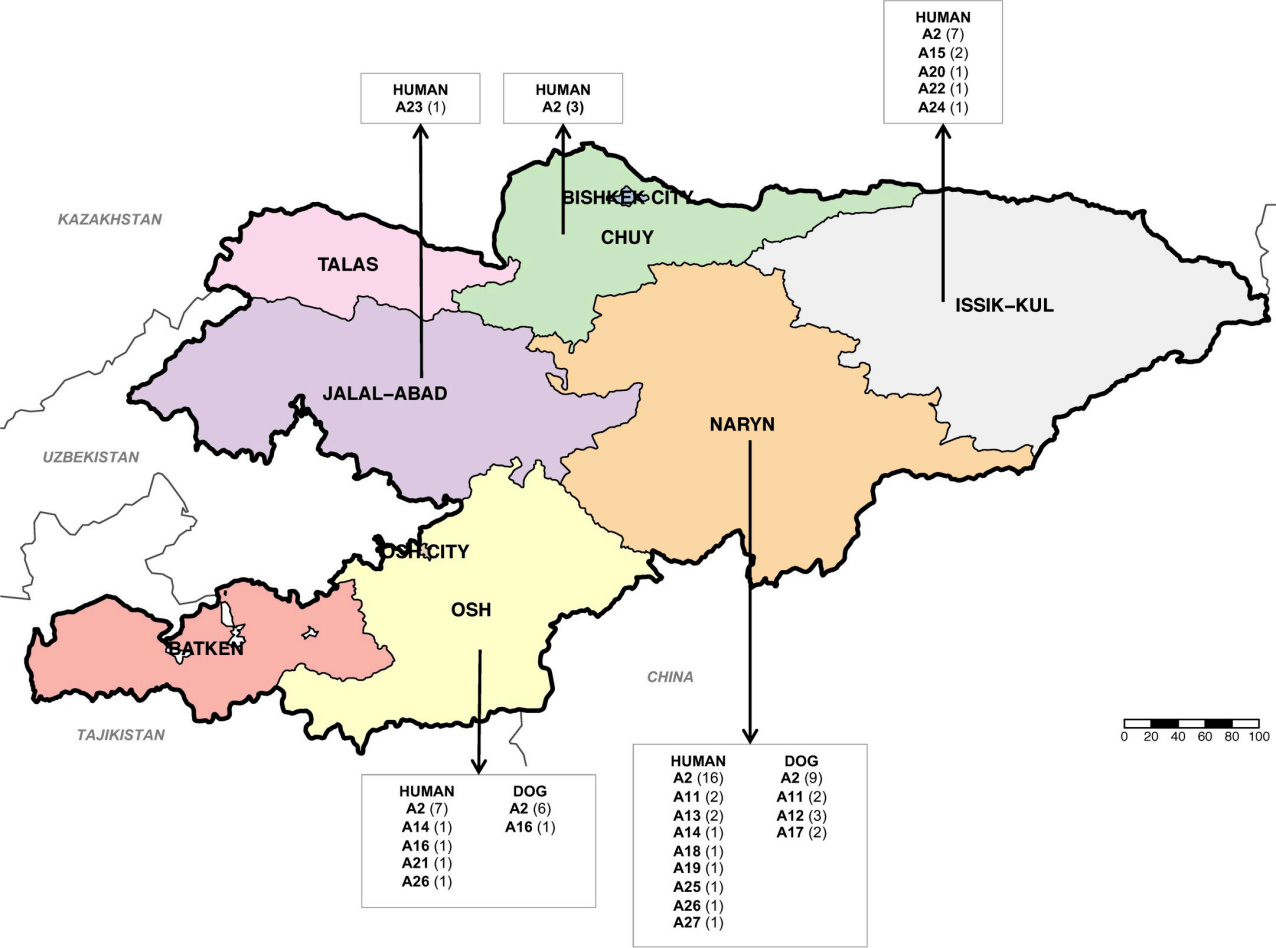
Map of Kyrgyzstan showing the region of origin of the *cob*/*nad2*/*cox1* haplotypes (A#) identified in samples of *E*. *multilocularis* analyzed in this study from humans and dogs. Haplotypes A1-A10 were previously identified by Nakao et al. 2009 [19] when the haplotypes A11 to A27 are from this study. The number of samples belonging to each haplotype is shown between brackets. Map source: http://www.diva-gis.org/gdata.

**Fig 2:**
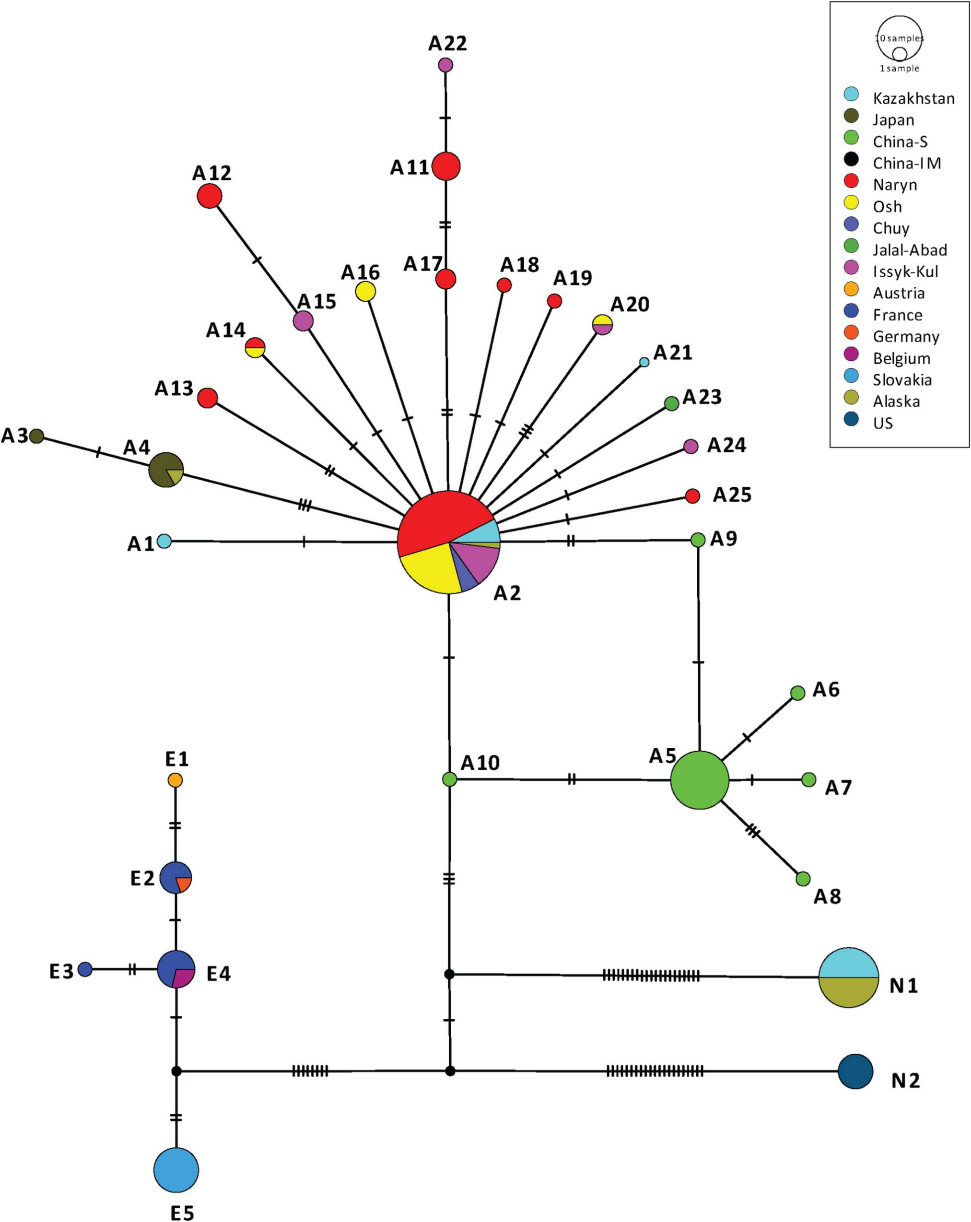
Haplotype network of the concatenated sequences of the genes *cob*, *nad2* and *cox1* from 75 isolates from *E*. *multilocularis* from this study including 52 samples from humans and 23 samples from dogs. Concatenated sequences from European haplotypes E1-E5, Asian haplotypes A1-A10 and North American haplotypes N1-N2 are also included; these sequences were downloaded from NCBI and were originally published by Nakao et al., [19]. Haplotypes described in this study are named A11-A26 (Accession Numbers: MN829497-MN829544); haplotype A26 is not included in the figure due to a nucleotide deletion in the *cox1* gene as explained in the results section. Nucleotide diversity n = 0.00333811, number of segregating sites = 83, number of parsimony-informative sites = 33, Tajima’s D: -0.611996 p (D<-0.611996) = 0.579195.

PCR confirmed the presence of *E*. *multilocularis* in 60 out of 61 AE human patients diagnosed in Kyrgyzstan. The remnant sample was additionally confirmed as AE using immunohistochemichal-stainings with monoclonal antibodies [26]. From the 60 samples mentioned above, it was possible to amplify and acquire good quality sequences from all the three genes of interest (*cob*, *nad2* and *cox1*) in 52 isolates. From the 28 canine faecal samples identified positive for *E*. *multilocularis*, it was possible to amplify and sequence the same three genes in 23 samples. Therefore, the concatenated sequences of the *cob*, *nad2* and *cox1* genes from 75 DNA isolates of *E*. *multilocularis* (52 human and 23 dogs) were used for further haplotype analysis. In total, 17 different *cob*/*nad2*/*cox1* haplotypes of *E*. *multilocularis* were identified in these 75 samples. When compared with the number of haplotypes using individual genes, the numbers of haplotypes decreased to 7 using only *cob*, 6 with *nad2* and 9 with *cox1*. Forty-eight isolates representing 64% of the total number of samples, were identified as the haplotype A2, originally described by Nakao et al [19] from four samples from Kazakhstan and one from St. Lawrence Island (Alaska, USA). Within the human samples, the haplotype A2 was identified in 63.4% (33 out of 52 isolates). This haplotype was identified in 65.2% (15 out of 23 isolates) from dogs. The other twenty-seven concatenated *cob/nad2/cox1* sequences were assigned to 16 not previously described haplotypes which were arbitrarily named A11-A26 (accession Numbers: MN829497-MN829544) following the nomenclature given by Nakao et al. [19] for isolates from Asia. The distribution of the 17 *cob*/*nad2*/*cox1* haplotypes of *E*. *multilocularis* found in this study in Kyrgyzstan and the number of samples (from humans and dogs) identified with each haplotype is shown in Fig 1. The haplotype A2 is present in four out of five regions from where human *E*. *multilocularis* samples were available (Chuy, Issyk-Kul, Naryn and Osh) and is the most common haplotype infecting humans in each of these regions. From the Jalal-Abad region the single sample available was identified as the haplotype A23. Also, the haplotype A2 was found in 6 out of 7 dog samples from Osh and in 9 out of 16 dog samples from Naryn. The network of the haplotypes identified in this study is shown in Fig 2, including sequences previously described by Nakao et al. [19] from Europe, Asia and North America. It has the typical star-like shape with all haplotypes from Kyrgyzstan clustering with the Asian group but assembling a different subgroup compared with the haplotypes previously described from Sichuan in China. The *cob*/*nad2*/*cox1* A2 haplotype is located in the central position for the Kyrgyzstan subgroup, while the haplotype *cob*/*nad2*/*cox1* A5 seems to be the central haplotype in Sichuan. Nucleotide substitutions of the mitochondrial *cob*, *nad2* and *cox1* genes in the 17 haplotypes of *E*. *multilocularis* are shown in S1 Table, S2 Table and S3 Table, respectively. The haplotype A26 has 2 deletions in the sequence of the *cox1* gene (positions 209 and 1,402) which produce internal stop codons, suggesting this is a pseudogene, and is not included in Fig 2. Table 1 shows values for genetic distance between subpopulations of *E*. *multilocularis* haplotypes identified in 3 regions of Kyrgyzstan where samples were available (Jalal-Abad and Chuy were excluded due to the low number of samples). Based on the values of pairwise fixation index (Fst), it is possible to say that there is no genetic differentiation between the regional parasite subpopulations found in Kyrgyzstan. Also, a low Fst value (-0.0494) was found when comparing the parasite populations of Kyrgyzstan and from the neighbouring country Kazakhstan (Table 2). However, Fst values near 1 were found when the total population of *cob*/*nad2*/*cox1* haplotypes of *E*. *multilocularis* described in Kyrgyzstan were compared with haplotypes of the parasite described in Europe, Asia (Japan and Sichuan, China), and North America (including St. Lawrence Island, Alaska).

**Table 1:** Pairwise fixation index (Fst) among *E*. *multilocularis* subpopulations from different regions of Kyrgyzstan.

Region	Naryn	Osh	Issyk-Kul
Naryn			
Osh	0.03490		
Issyk-Kul	-0.01032	0.03015	-

(Fst values nearing 1 indicate extreme genetic differentiation between two subpopulations). Significant values are indicated by an asterisk (p < 0.05).

**Table 2:** Pairwise fixation index (Fst) among *E*. *multilocularis* subpopulations from Kyrgyzstan and Kazakhstan, China (CHN-S Sichuan), Japan, St. Lawrence Island (Alaska, USA), Europe (Austria, France, Germany, Belgium and Slovakia), and North America (Indiana and South Dakota).

	Kazakhstan	CHN-S	Japan	St Law	Europe	North America
Kyrgyzstan	-0.04940	0.69294*	0.69556*	0.88180*	0.89771*	0.95316*

Fst values nearing 1 indicate extreme genetic differentiation between two subpopulations. Significant values are indicated by an asterisk (p < 0.05).

### *E*. *granulosus s*.*l.*

**Fig 3:**
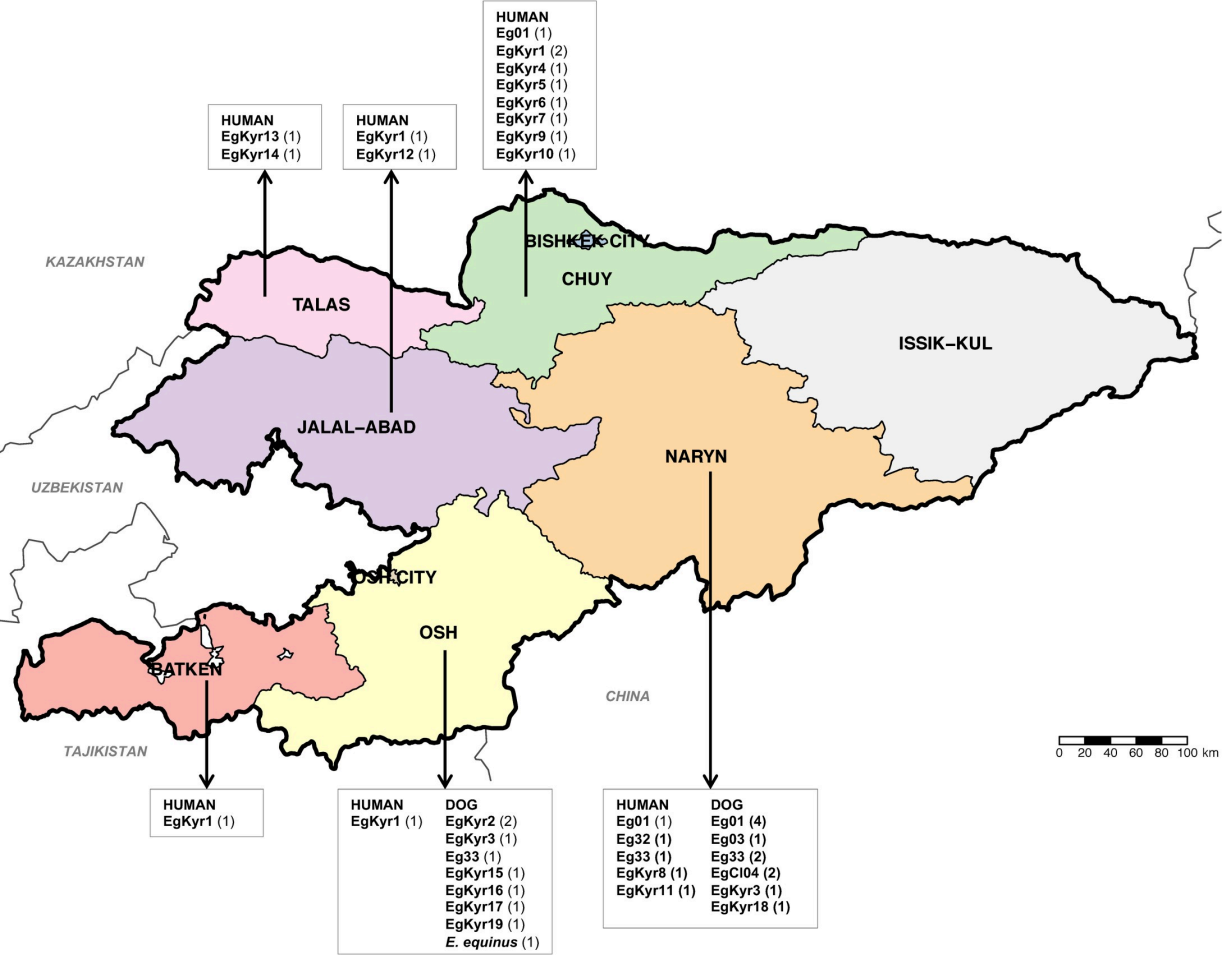
Map of Kyrgyzstan showing the region of origin of the *cox1* haplotypes (Eg#) identified in samples of *E*. *granulosus s*.*l*. from humans and dogs in this study. The number of samples belonging to each haplotype is shown in brackets. Map source: http://www.diva-gis.org/gdata.

**Fig 4:**
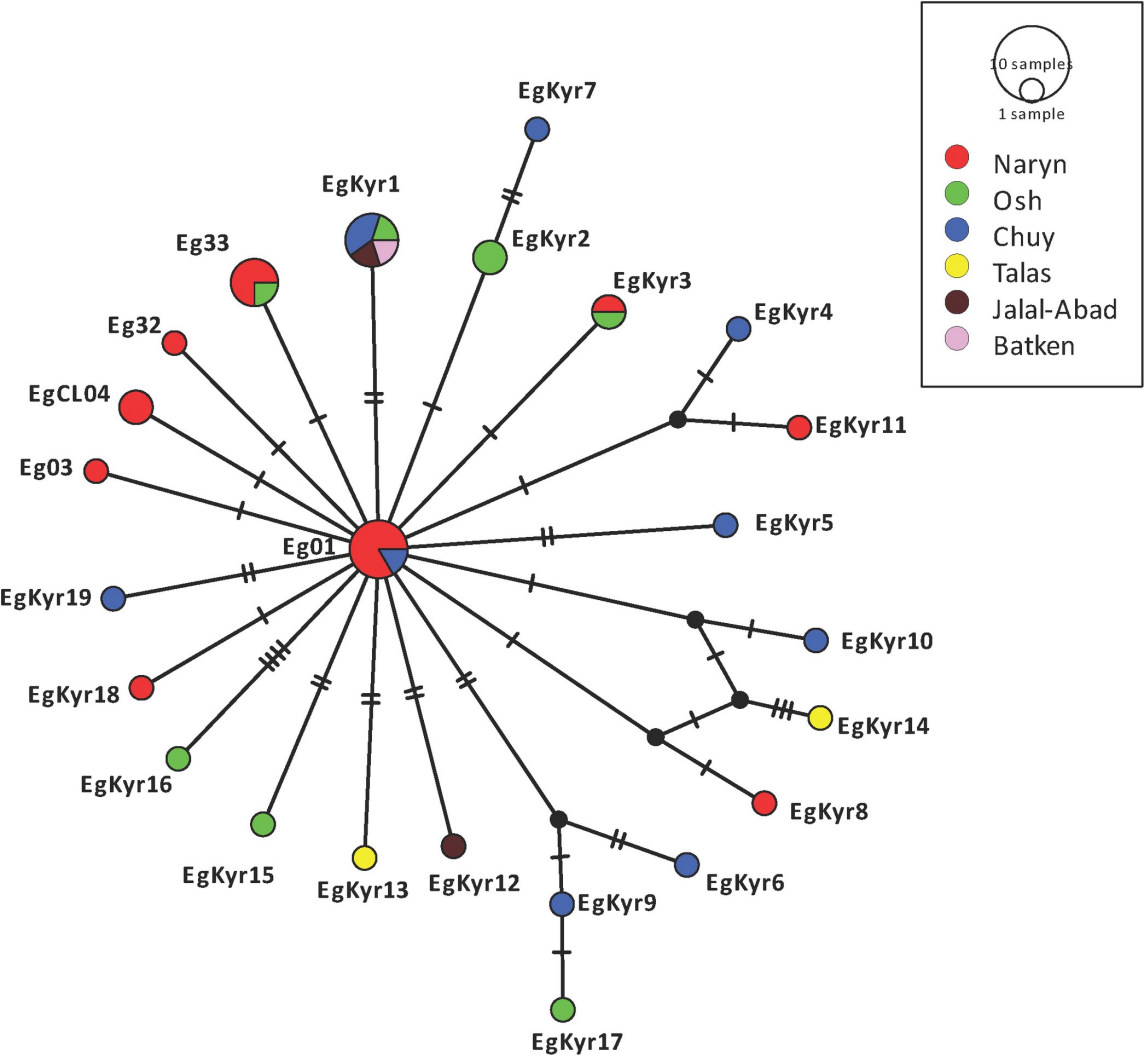
Haplotype network of the sequences of the *cox1* gene of the 39 isolates from *E*. *granulosus s*.*s*. included in this study including 20 samples from humans and 19 samples from dogs. Haplotypes described in this study include EgKyr1-EgKyr19 (Accession numbers MN787537-MN787561). Sequences from haplotypes previously described were also included: Eg01 (JQ250806), Eg03 (JQ250808), Eg32 (AB688609), Eg33 (AB688610) and EgCl04 (KX227119). Nucleotide diversity n = 0.00197019, number of segregating sites = 40, number of parsimony-informative sites = 7, Tajima’s D: -2.34668 p (D<-2.34668) = 0.00351151.

It was possible to amplify the *cox1* gene in 20 out of the 23 human CE samples and in 20 out of 24 canine faecal samples positive for *E*. *granulosus s*.*l*. In total, 24 samples have a sequence 100% homologous with the original description of G1 (366bp of the *cox1*) by Bowles et al, 1992 [25]. While fifteen other samples have a sequence representing nine different genotypes which differ between one and three nucleotides with the G1 and G3 sequences therefore were identified as *E*. *granulosus s*.*s*. Finally, one isolate was identified as *E*. *equinus* (G4). A total of 24 different *cox1* haplotypes of *E*. *granulosus s*.*s*. were identified from 39 *cox1* sequences while a single sequence from a dog was identified as *E*. *equinus*. The sequence identified as *E*. *equinus* sequence (MN787562) shows 100% homology with isoales from Turkey (KY766905) and the United Kingdom (AB786665). Six samples (two from human and four from dogs), equivalent to 15.4% of the samples, were identified as Eg01 (JQ250806) which is the most commonly distributed *cox1* haplotype of *E*. *granulosus s*.*s*. worldwide. Four samples (one human and three from dogs) were identified as Eg33 (AB688610), and one human sample was identified as the haplotype Eg32 (AB688609), both haplotypes previously described in China. Two samples from dogs were identified as EgCl04 (KX227119) initially described in Chile and another sample from a dog was identified as Eg03 (JQ250808) initially described in Iran and Jordan. The twenty-five remnant sequences were identified as 19 not previously described *cox1* haplotypes of *E*. *granulosus s*.*s*. (named EgKyr1 to EgKyr19, accession numbers MN787537-MN787561). From these “new” haplotypes the most frequent was EgKyr1 present in 5 human samples. The distribution of the 24 *cox1* haplotypes of *E*. *granulosus s*.*s*. and the single *E*. *equinus* sample found in this study in Kyrgyzstan and the number of samples (from humans and dogs) identified with particular haplotypes is shown in Fig 3. The most common and cosmopolitan *cox1* haplotype, Eg01, was found in the provinces of Chuy and Naryn regions, but it was only in Naryn that this haplotype was found in both humans and dogs. The *cox1* haplotype EgKyr1 is present in four out of six districts from where human samples were available (1 from Osh, Batken and Jalal-Abad and 2 from Chuy) (Fig 3). The haplotypes EgCl04 and Eg03 were found only in Naryn. The haplotype network built with the sequences of isolates from this study is shown in Fig 4. A typical star-like shape is observed with the Eg01 haplotype in the centre of the network and the other haplotypes from Kyrgyzstan differing between 1 and 8 nucleotides with the Eg01 sequence. Nucleotide substitutions of mitochondrial *cox1* gene in the 24 haplotypes of *E*. *granulosus s*.*s*. identified in this study are shown in S4 Table. Similarly to *E*. *multilocularis*, based on Fst values it is possible to say that there is no genetic differentiation between the parasite subpopulations found within Kyrgyzstan for *E*. *granulosus s*.*s*. (Batken region was excluded due to the low number of samples) (Table 3).

**Table 3:** Pairwise fixation index (Fst) among *E*. *granulosus s*.*s*. subpopulations from different provinces of Kyrgyzstan.

Region	Naryn	Osh	Chuy	Talas
Naryn				
Osh	0.04860			
Chuy	0.04167	0.00316		
Talas	0.08754	0.01999	0.01999	
Jalal-Abad	0.08754	-0.03796	-0.10318	0.00000

Fst values nearing 1 indicate extreme genetic differentiation between two subpopulations. Significant values are indicated by an asterisk (p < 0.05).

## Discussion

The study of genetic variability in *Echinococcus* species has made a significant contribution to the knowledge of epidemiology, geographic distribution and phylogeny of these parasites. Since early studies in the 90’s it was clear that a higher degree of variability within *E*. *granulosus s*.*l*. was present compared with *E*. *multilocularis* [25,27]. Subsequent studies on the genetic variability of isolates causing CE have contributed clarifying the taxonomy of the parasite, grouping a number of species under the complex *E*. *granulosus s*.*l*. In the case of *E*. *multilocularis* the sequencing of the full length of three mitochondrial genes (*cob*, *cox1* and *nad2*) clearly identified different haplotypes of the parasite clustering in European, Asian and North American clades [19]. Genetic variability within *E*. *multilocularis* has also been extensively studied using microsatellite markers [28,29,30]. Although it is also possible to distinguish between Asian, European and North American clades, to date, there is no study discerning a possible connection between mitochondrial and microsatellite markers except for a conference paper in which no obvious correlation was found between individual EmsB profiles and certain mitochondrial haplotypes (*cox1*, *nd1* and *atp6* genes) [31]. Using the methodology described by Nakao et al. [19] and Yanagida et al. [24] for the analysis of genetic variability within *E*. *multilocularis* and *E*. *granulosus s*.*s*., respectively, allowed us to produce datasets which are comparable with other sequences from different geographic regions. In total, 52 isolates from AE patients and 20 from CE patients were included in the study. In the case of *E*. *multilocularis*, Nakao et al [19] included only 5 human samples (from Sichuan, China) together with 24 samples from rodents, 6 from red foxes and 2 from dogs. The small number of samples from humans might reflect the difficulty in acquiring such parasite material. Other investigations which have included human samples have sequenced shorter sections of the mitochondrial genes [32] or the full length of different mitochondrial genes [33]. Therefore, we cannot compare our dataset with such sequences. For *E*. *granulosus*, the analysis of genetic diversity using metacestode material derived from humans has been more extensively used [34] but mostly using short sequences of mitochondrial genes.

The *cob*/*nad2*/*cox1* haplotype A2 of *E*. *multilocularis*, originally described in 4 isolates from Kazakhstan and in one isolate from St Lawrence island (Alaska, USA) [19], was the predominant *E*. *multilocularis* haplotype found in the human and dog samples in this study. Based on the finding of A2 and the haplotype A4 (in Japan and St. Lawrence Island), it was hypothesized that the long-distance dispersal of *E*. *multilocularis* in the Asian continent occurred during the Holocene to the present. Interestingly, the central location of the A2 haplotype in the network described in this study (Fig 2) suggests that this is an ancient haplotype from which other variants of the parasite have mutated. However, it is important to clarify that what is considered to be a *cob*/*nad2*/*cox* A2 haplotype could differ in the sequence in other mitochondrial genes. To confirm if all the sequences called A2 (by Nakao et al. [19] and in this study) are actually the same variant of the parasite it is necessary to sequence the whole mitochondrial genome of such isolates, however, this is not the objective of the present study. In fact, recent publications of nearly complete mitochondrial genome of different isolates of *E*. *granulosus s*.*s*. have shown that, in some cases, what was supposed to be the same haplotype (for example based on the sequence of the *cox1* gene only) in reality corresponded to different variants of the parasite when the whole mitogenome is sequenced [35]. Nevertheless, the identification of a single *cob*/*nad2*/*cox1* haplotype of *E*. *multilocularis* in this study, as the most common variant of the parasite in humans (63.4%) and dogs (65.2%) from Kyrgyzstan is relevant to understand the transmission of the parasite to humans. This haplotype was not found in the Jalal-Abad region, however we had only one isolate from this region. Jalal-Abad is the region with the lowest incidence of AE in Kyrgyzstan and this explains the low number of samples from this area [8] and this explains the low number of samples from this area. It would be interesting to perform similar studies in *E*. *multilocularis* isolated from foxes in the same country to know if this is also the most common variant of the parasite in the wild animal cycle. Also, it would be interesting to study more human samples in Asia to see if the A2 haplotype is responsible for most human infections in the continent. It is possible to speculate that the emergence of echinococcosis in Kyrgyzstan could be partially attributed to a specific variant(s) of the parasite circulating in the country. However, there is no evidence which attributes differences in virulence or host/parasite interaction to specific mitochondrial haplotypes of *E*. *multilocularis*. A similar situation occurs describing the genetic variability within *E*. *granulosus s*.*s*. The sequencing of the full nuclear genome from isolates identified as different mitochondrial haplotypes of *Echinococcus* species could be useful information to better understand if different haplotypes vary in the sequence of important genes which allow the establishment and survival of the parasites at their different stages. Interestingly, the haplotypes of *E*. *multilocularis* found in this study differ from the ones described in neighbouring China, specifically from Sichuan and Inner Mongolia, clearly reflecting differences in the parasite population (Table 2). Such differences may have been facilitated by the physical barrier which separates China and Kyrgyzstan. This border is 1,063km in length with various mountain ridges and peaks of the Tian Shan mountain system, some of them reaching over 7,000 m and also includes the Turpan Depression 154 m below sea level which is also one of the hottest and driest areas in China during the summer. The endemic areas of Sichuan and Inner Mongolia are over 1,000 km east of these geographical barriers. However, there is no support for the differentiation of the parasite population found within the country in the studied regions (Table 1).

Regarding the analysis of *E*. *granulosus s*.*s*. samples, we found a high variability identifying 19 not previously described *cox1* haplotypes. Interestingly, the most common and cosmopolitan *cox1* haplotype of *E*. *granulosus s*.*s*. (Eg01) was described only in six samples from Chuy (one human sample) and Naryn (one human and four dog samples) regions. The most common *cox1* haplotype infecting humans in Kyrgyzstan is EgKyr1 in 5 isolates from Osh, Batken, Jalal-Abad and Chuy. Unlike the case of *E*. *multilocularis*, there is no dominant haplotype of *E*. *granulosus* in the samples analysed (Fig 4). This could be the consequence of a low number of samples analysed or the higher variability of the sequences investigated. It is likely that the geographic isolation of Kyrgyzstan has allowed the parasite to mutate differently (at least in the *cox1* gene), compared with other parts of the world. Fst values do not support the differentiation of the population of *E*. *granulosus s*.*s*. within the country (Table 3). After multiple investigations of genetic variability within *E*. *granulosus s*.*s*. (see [34,36]) it remains unknown if the variants of this parasite described (based on the sequence of the *cox1* gene) are actually different in terms of pathogenicity, biological features and host response. In this study, we did not find any correlation between a specific haplotype and classification or size of the lesion. In the meantime, data from this study provides valuable information regarding the phylogeography and distribution of the parasite. Interestingly, we did not find other species than *E*. *granulosus s*.*s*. and *E*. *equinus*, although previous research has also identified *E*. *intermedius* G6/7 in dogs from Kyrgyzstan [13] and South-East Kazakhstan, close to the border with Kyrgyzstan [16].

In summary, we described that the *cob*/*nad2*/*cox1* A2 haplotype of *E*. *multilocularis* is the most commonly found variant of the parasite in humans and dogs in Kyrgyzstan. Its central location in the haplotype network built here suggests that it is an ancient variant of the parasite. In the case of *E*. *granulosus s*.*s*. there is no dominant *cox1* haplotype in the samples analysed, however a number of non previously described haplotypes have been characterized. Further investigations should clarify if the A2 haplotype is also the most relevant variant infecting humans in other countries in Asia.

## Supporting information

S1 Table Segregating sites between the concatenated sequences of the *cob* gene of the haplotypes of *E*. *multilocularis* identified in this study (haplotypes A11 to A26) compared with the sequence of the already described haplotypes of E. multilocularis by Nakao et al., 2009 [19] (haplotypes A1-A10 excluding O1). (DOCX)

S2 Table Segregating sites between the concatenated sequences of the *nad2* gene of the haplotypes of *E*. *multilocularis* identified in this study (haplotypes A11 to A26) compared with the sequence of the already described haplotypes of *E*. *multilocularis* by Nakao et al., 2009 [19] (haplotypes A1-A10 excluding O1). (DOCX)

S3 Table Segregating sites between the concatenated sequences of the *cox1* gene of the haplotypes of *E*. *multilocularis* identified in this study (haplotypes A11 to A26) compared with the sequence of the already described haplotypes of *E*. *multilocularis* by Nakao et al, 2009 (haplotypes A1-A10 excluding O1). (DOCX)

S4 Table Segregating sites between the sequences of the *cox1* gene of the haplotypes of *E*. *granulosus sensu stricto* identified in this study (haplotypes EgKyr1-EgKyr19) compared with the sequence of the already described haplotypes of *E*. *granulosus s*.*s*. Eg01 (JQ250806), Eg03 (JQ250808), Eg32 (AB688609), Eg33 (AB688610) and EgCl04 (KX227119) also present in Kyrgyzstan. (DOCX)

## References

[1] Eckert J, Deplazes P (2004) Biological, Epidemiological, and Clinical Aspects of Echinococcosis, a Zoonosis of Increasing Concern. Clinical Microbiology Reviews 17: 107–135. 10.1128/CMR.17.1.107-135.2004 14726458 PMC321468

[2] Deplazes P, Rinaldi L, Alvarez Rojas CA, Torgerson PR, Harandi MF, Romig T, et al (2017) Chapter Six—Global Distribution of Alveolar and Cystic Echinococcosis In: Thompson RCA, Deplazes P, Lymbery AJ, editors. Advances in Parasitology: Academic Press pp. 315–493. 10.1016/bs.apar.2016.11.001 28131365

[3] Bebezov B, Mamashev N, Umetaliev T, Ziadinov I, Craig PS, Joekel D, et al (2018) Intense Focus of Alveolar Echinococcosis, South Kyrgyzstan. Emerging infectious diseases 24: 1119–1122. 10.3201/eid2406.161641 29774832 PMC6004877

[4] Torgerson PR (2013) The emergence of echinococcosis in central Asia. Parasitology 140: 1667–1673. 10.1017/S0031182013000516 23659353

[5] Abdybekova A, Sultanov A, Karatayev B, Zhumabayeva A, Shapiyeva Z, Yeshmuratov T, et al (2015) Epidemiology of echinococcosis in Kazakhstan: an update. Journal of Helminthology 89: 647–650. 10.1017/S0022149X15000425 26160276

[6] Raimkylov KM, Kuttubaev OT, Toigombaeva VS (2015) Epidemiological analysis of the distribution of cystic and alveolar echinococcosis in Osh Oblast in the Kyrgyz Republic, 2000–2013. J Helminthol 89: 651–654. 10.1017/S0022149X15000565 26442705 PMC4700905

[7] Usubalieva J, Minbaeva G, Ziadinov I, Deplazes P, Torgerson PR (2013) Human alveolar echinococcosis in Kyrgyzstan. Emerg Infect Dis 19: 1095–1097. 10.3201/eid1907.121405 23763935 PMC3713972

[8] Paternoster G, Boo G, Wang C, Minbaeva G, Usubalieva J, Raimkulov KM, et al (2020) Epidemic cystic and alveolar echinococcosis in Kyrgyzstan: an analysis of national surveillance data. The Lancet Global Health 8: e603–e611. 10.1016/S2214-109X(20)30038-3 32199126

[9] Counotte MJ, Minbaeva G, Usubalieva J, Abdykerimov K, Torgerson PR (2016) The Burden of Zoonoses in Kyrgyzstan: A Systematic Review. PLoS Negl Trop Dis 10: e0004831 10.1371/journal.pntd.0004831 27387925 PMC4936671

[10] Budke CM, Deplazes P, Torgerson PR (2006) Global socioeconomic impact of cystic echinococcosis. Emerg Infect Dis 12: 296–303. 10.3201/eid1202.050499 16494758 PMC3373106

[11] Torgerson PR, Ziadinov I, Aknazarov D, Nurgaziev R, Deplazes P (2009) Modelling the age variation of larval protoscoleces of *Echinococcus granulosus* in sheep. International Journal for Parasitology 39: 1031–1035. 10.1016/j.ijpara.2009.01.004 19504758

[12] Van Kesteren F, Mastin A, Mytynova B, Ziadinov I, Boufana B, Torgerson P, et al (2013) Dog ownership, dog behaviour and transmission of *Echinococcus* spp. in the Alay Valley, southern Kyrgyzstan. Parasitology 140: 1674–1684. 10.1017/S0031182013001182 23985326 PMC3806042

[13] Ziadinov I, Mathis A, Trachsel D, Rysmukhambetova A, Abdyjaparov TA, Kuttubaev OT, et al (2008) Canine echinococcosis in Kyrgyzstan: using prevalence data adjusted for measurement error to develop transmission dynamics models. Int J Parasitol 38: 1179–1190. 10.1016/j.ijpara.2008.01.009 18371969 PMC2527539

[14] Ziadinov I, Deplazes P, Mathis A, Mutunova B, Abdykerimov K, Nurgaziev R, et al (2010) Frequency distribution of *Echinococcus multilocularis* and other helminths of foxes in Kyrgyzstan. Veterinary Parasitology 171: 286–292. 10.1016/j.vetpar.2010.04.006 20434845 PMC2903646

[15] Afonso E, Knapp J, Tête N, Umhang G, Rieffel D, van Kesteren F.et al (2015) *Echinococcus multilocularis* in Kyrgyzstan: similarity in the Asian EmsB genotypic profiles from village populations of Eastern mole voles (*Ellobius tancrei*) and dogs in the Alay valley. J Helminthol 89: 664–670. 10.1017/S0022149X15000474 26137938 PMC4700906

[16] Trachsel D, Deplazes P, Mathis A (2007) Identification of taeniid eggs in the faeces from carnivores based on multiplex PCR using targets in mitochondrial DNA. Parasitology 134: 911–920. 10.1017/S0031182007002235 17288631

[17] Mathis A, Deplazes P, Eckert J (1996) An improved test system for PCR-based specific detection of *Echinococcus multilocularis* eggs. J Helminthol 70: 219–222. 10.1017/s0022149x00015443 8960218

[18] Štefanić S, Shaikenov BS, Deplazes P, Dinkel A, Torgerson PR, Mathis A (2004) Polymerase chain reaction for detection of patent infections of *Echinococcus granulosus* (“sheep strain”) in naturally infected dogs. Parasitology Research 92: 347–351. 10.1007/s00436-003-1043-y 14727186

[19] Nakao M, Xiao N, Okamoto M, Yanagida T, Sako Y, Ito A, (2009) Geographic pattern of genetic variation in the fox tapeworm *Echinococcus multilocularis*. Parasitology International 58: 384–389. 10.1016/j.parint.2009.07.010 19651237

[20] Nakao M, Sako Y, Ito A (2003) Isolation of polymorphic microsatellite loci from the tapeworm *Echinococcus multilocularis*. Infection, Genetics and Evolution 3: 159–163. 10.1016/s1567-1348(03)00070-4 14522179

[21] Clement M, Posada D, Crandall KA (2000) TCS: a computer program to estimate gene genealogies. Molecular Ecology 9: 1657–1659. 10.1046/j.1365-294x.2000.01020.x 11050560

[22] Leigh JW, Bryant D (2015) popart: full-feature software for haplotype network construction. Methods in Ecology and Evolution 6: 1110–1116.

[23] Excoffier L, Lischer HEL (2010) Arlequin suite ver 3.5: a new series of programs to perform population genetics analyses under Linux and Windows. Molecular Ecology Resources 10: 564–567. 10.1111/j.1755-0998.2010.02847.x 21565059

[24] Yanagida T, Mohammadzadeh T, Kamhawi S, Nakao M, Sadjjadi SM, Hijjawi N, et al (2012) Genetic polymorphisms of *Echinococcus granulosus sensu stricto* in the Middle East. Parasitology International 61: 599–603. 10.1016/j.parint.2012.05.014 22668837

[25] Bowles J, Blair D, McManus DP (1992) Genetic variants within the genus *Echinococcus* identified by mitochondrial DNA sequencing. Molecular and Biochemical Parasitology 54: 165–173. 10.1016/0166-6851(92)90109-w 1435857

[26] Reinehr M, Micheloud C, Grimm F, Kronenberg PA, Grimm J, Beck A, et al (2020) Pathology of Echinococcosis: A Morphologic and Immunohistochemical Study on 138 Specimens With Focus on the Differential Diagnosis Between Cystic and Alveolar Echinococcosis. The American Journal of Surgical Pathology 44: 43–54. 10.1097/PAS.0000000000001374 31567204

[27] Bowles J, McManus DP (1993) NADH dehydrogenase 1 gene sequences compared for species and strains of the genus *Echinococcus*. International Journal for Parasitology 23: 969–972. 10.1016/0020-7519(93)90065-7 8106191

[28] Bretagne S, Robert B, Vidaud D, Goossens M, Houin R (1991) Structure of the *Echinococcus multilocularis* U1 snRNA gene repeat. Molecular and Biochemical Parasitology 46: 285–292. 10.1016/0166-6851(91)90052-8 1840625

[29] Bretagne S, Assouline B, Vidaud D, Houin R, Vidaud M (1996) *Echinococcus multilocularis*: Microsatellite Polymorphism in U1 snRNA Genes. Experimental Parasitology 82: 324–328. 10.1006/expr.1996.0040 8631384

[30] Knapp J, Bart JM, Glowatzki ML, Ito A, Gerard S, Maillard S, et al (2007) Assessment of Use of Microsatellite Polymorphism Analysis for Improving Spatial Distribution Tracking of *Echinococcus multilocularis*. Journal of Clinical Microbiology 45: 2943–2950. 10.1128/JCM.02107-06 17634311 PMC2045259

[31] Schroer S, Knapp J, Gottstein B, Dinkel A, Romig T. Genetic diversity of *Echinococcus multilocularis*–comparative results from mitochondrial and microsatellite markers. In: Dominique A. Vuitton LM, Bruno Gottstein and Patrick Giraudoux, editor; 2014; Besancon, France.

[32] Wu C, Zhang W, Ran B, Fan H, Wang H, Guo B, et al (2017) Genetic variation of mitochondrial genes among *Echinococcus multilocularis* isolates collected in western China. Parasites & Vectors 10: 265. 28558809 10.1186/s13071-017-2172-y PMC5450100

[33] Shang J, Zhang G, Yu W, He W, Wang Q, Zhong B, et al (2019) Molecular characterization of human echinococcosis in Sichuan, Western China. Acta Tropica 190: 45–51. 10.1016/j.actatropica.2018.09.019 30278154

[34] Alvarez Rojas CA, Romig T, Lightowlers MW (2014) *Echinococcus granulosus sensu lato* genotypes infecting humans–review of current knowledge. International Journal for Parasitology 44: 9–18. 10.1016/j.ijpara.2013.08.008 24269720

[35] Kinkar L, Laurimäe T, Sharbatkhori M, Mirhendi H, Kia EB, Ponce-Gordo F, et al (2017) New mitogenome and nuclear evidence on the phylogeny and taxonomy of the highly zoonotic tapeworm *Echinococcus granulosus sensu stricto*. Infection, Genetics and Evolution 52: 52–58. 10.1016/j.meegid.2017.04.023 28456662

[36] Romig T, Ebi D, Wassermann M (2015) Taxonomy and molecular epidemiology of *Echinococcus granulosus sensu lato*. Veterinary Parasitology 213: 76–84. 10.1016/j.vetpar.2015.07.035 26264250

