# Supplementary material for: Genetic diversity of Echinococcus multilocularis and Echinococcus granulosus sensu lato in Kyrgyzstan: The A2 haplotype of E. multilocularis is the predominant variant infecting humans
Source: PLoS Negl Trop Dis. 2020 May 13;14(5):e0008242. doi: 10.1371/journal.pntd.0008242 (PMC7219741; doi:10.1371/journal.pntd.0008242)
Supplement: S2 Table — (DOCX) [file pntd.0008242.s002.docx]

| Nucleotide position | | | | | | | | | | | | | | | | | | | | | | |
| --- | --- | --- | --- | --- | --- | --- | --- | --- | --- | --- | --- | --- | --- | --- | --- | --- | --- | --- | --- | --- | --- | --- |
|  | 16 | 42 | 58 | 72 | 123 | 157 | 158 | 229 | 234 | 246 | 252 | 338 | 346 | 393 | 423 | 486 | 633 | 656 | 717 | 786 | 796 | 827 |
| A1  A2  A3  A4  A5  A6  A7  A8  A9  A10  A11  A12  A13  A14  A15  A16  A17  A18  A19  A20  A21  A22  A23  A24  A25  A26  E1  E2  E3  E4  E5  N1  N2 | G  .  A  A  .  .  .  .  .  .  .  .  .  .  .  .  .  .  .  .  .  .  .  .  .  .  .  .  .  .  .  .  . | G  .  .  .  .  .  .  .  .  .  .  .  .  .  .  .  .  .  .  .  .  .  .  .  .  .  T  T  T  T  T  .  . | T  .  .  .  .  .  .  .  .  .  .  .  .  .  .  .  .  .  .  .  .  G  .  .  .  .  .  .  .  .  .  .  . | G  .  .  .  .  .  .  .  .  .  .  .  .  .  .  .  .  .  .  .  .  .  .  .  .  .  A  A  .  .  .  .  . | T  .  .  .  .  .  .  .  .  .  .  .  .  .  .  .  .  .  .  .  .  .  .  .  .  .  C  C  C  C  C  C  C | G  .  .  .  .  .  .  .  .  .  .  .  .  .  .  .  .  .  .  .  .  .  .  .  .  .  .  .  .  .  .  .  T | C  .  .  .  .  .  .  .  .  .  T  .  .  .  .  .  T  .  .  .  .  T  .  .  .  .  .  .  .  .  .  .  . | C  .  .  .  .  .  .  .  .  .  .  .  .  .  .  .  .  .  .  .  .  .  T  .  .  .  .  .  .  .  .  .  . | A  .  .  .  .  .  .  .  .  .  .  .  .  .  .  .  .  .  .  .  .  .  .  .  .  .  .  .  .  .  .  .  G | A  .  .  .  .  .  .  .  .  .  .  .  .  .  .  .  .  .  .  .  .  .  .  .  .  .  .  .  .  .  .  G  G | A  .  .  .  .  .  .  .  .  .  .  .  .  .  .  .  .  .  .  .  .  .  .  .  .  .  T  T  T  T  T  .  . | G  .  T  T  .  .  .  .  .  .  .  .  .  .  .  .  .  .  .  .  .  .  .  .  .  .  .  .  .  .  .  .  . | A  .  .  .  .  .  .  .  .  .  .  .  .  .  .  G  .  .  .  .  .  .  .  .  .  .  .  .  .  .  .  .  . | G  .  .  .  .  .  .  .  .  .  .  .  .  .  .  .  .  .  .  T  .  .  .  .  .  .  .  .  .  .  .  A  . | T  .  .  .  .  .  .  .  .  .  .  .  .  .  .  .  .  .  .  .  .  .  .  .  .  .  C  C  C  C  C  A  C | A  .  .  .  .  .  .  .  .  .  .  .  .  .  .  .  .  .  .  .  .  .  .  .  .  .  .  .  .  .  G  .  . | T  .  .  .  .  .  .  .  .  .  .  .  .  .  .  .  .  .  .  .  .  .  .  .  .  .  .  .  .  .  .  G  . | C  .  .  .  T  T  T  T  T  .  .  .  .  .  .  .  .  .  .  .  .  .  .  .  .  .  .  .  .  .  .  .  . | G  .  A  A  .  .  .  .  .  .  .  .  .  .  .  .  .  .  .  .  .  .  .  .  .  .  .  .  .  .  .  .  . | G  .  .  .  .  .  .  .  .  .  .  .  .  .  .  .  .  .  .  .  .  .  .  .  .  .  .  .  .  .  .  A  . | A  .  .  .  .  .  .  .  .  .  .  .  .  .  .  .  .  .  .  .  .  .  .  .  .  .  .  .  .  .  .  .  G | T  .  .  .  A  A  A  A  A  .  .  .  .  .  .  .  .  .  .  .  .  .  .  .  .  .  .  .  .  .  .  .  . |

Supplementary Table 2 Segregating sites between the concatenated sequences of the *nad2* gene of the haplotypes of *E. multilocularis* identified in this study (haplotypes A11 to A26) compared with the sequence of the already described haplotypes of *E. multilocularis* by Nakao et al, 2009 (haplotypes A1-A10 excluding O1). Nucleotide positions are numbered from the first nucleotide of the gene.
