# Supplementary material for: Genetic diversity of Echinococcus multilocularis and Echinococcus granulosus sensu lato in Kyrgyzstan: The A2 haplotype of E. multilocularis is the predominant variant infecting humans
Source: PLoS Negl Trop Dis. 2020 May 13;14(5):e0008242. doi: 10.1371/journal.pntd.0008242 (PMC7219741; doi:10.1371/journal.pntd.0008242)
Supplement: S3 Table — (DOCX) [file pntd.0008242.s003.docx]

| Nucleotide position | | | | | | | | | | | | | | | | | | | | | | | | | | | | | | | | |
| --- | --- | --- | --- | --- | --- | --- | --- | --- | --- | --- | --- | --- | --- | --- | --- | --- | --- | --- | --- | --- | --- | --- | --- | --- | --- | --- | --- | --- | --- | --- | --- | --- |
|  | 42 | 199 | 212 | 257 | 289 | 336 | 346 | 364 | 368 | 398 | 420 | 454 | 585 | 643 | 675 | 688 | 721 | 735 | 760 | 800 | 822 | 926 | 1048 | 1194 | 1293 | 1314 | 1329 | 1351 | 1388 | 1399 | 1495 | 1573 |
| A1  A2  A3  A4  A5  A6  A7  A8  A9  A10  A11  A12  A13  A14  A15  A16  A17  A18  A19  A20  A21  A22  A23  A24  A25  A26  E1  E2  E3  E4  E5  N1  N2 | A  .  .  .  .  .  .  .  .  .  .  .  .  .  .  .  .  .  .  C  .  .  .  .  .  .  .  .  .  .  .  .  . | A  .  .  .  .  .  .  .  .  .  .  .  .  G  .  .  .  .  .  .  .  .  .  .  .  .  .  .  .  .  .  .  . | T  .  .  .  .  .  .  .  .  .  .  .  .  .  .  .  .  .  .  .  .  .  .  .  .  -  .  .  .  .  .  .  . | T  .  .  .  .  .  .  .  .  .  .  .  .  .  .  .  .  .  .  .  .  .  .  .  .  .  C  C  C  C  C  .  . | T  .  .  .  .  .  .  .  .  .  .  .  .  .  .  .  .  .  .  .  .  .  .  .  .  .  C  C  C  C  C  C  C | A  .  .  .  .  .  .  .  .  .  .  .  .  .  .  .  .  .  .  .  .  .  .  .  .  .  .  .  .  .  .  G  . | T  .  .  .  .  .  .  .  .  .  .  .  .  .  .  .  .  .  .  .  .  .  .  .  .  .  C  .  .  .  .  .  . | G  .  .  .  .  .  .  .  .  .  .  .  .  .  .  .  .  .  .  .  .  .  .  .  .  .  T  .  .  .  .  .  . | C  .  .  .  .  .  .  .  .  .  .  .  .  .  .  .  .  .  .  .  .  .  .  .  .  .  .  .  T  .  .  .  . | T  .  .  .  .  .  .  .  .  .  .  .  .  .  .  .  .  .  C  .  .  .  .  .  .  .  .  .  .  .  .  .  . | G  .  .  .  .  .  .  .  .  .  .  .  A  .  .  .  .  .  .  .  .  .  .  .  .  .  .  .  .  .  .  .  . | C  .  .  .  .  .  .  .  .  .  .  .  .  .  .  .  .  .  .  T  .  .  .  .  .  .  .  .  .  .  .  .  . | G  .  .  .  .  .  .  .  .  .  .  .  .  .  .  .  .  .  .  .  .  .  .  .  .  .  .  .  .  .  .  .  A | T  .  .  .  .  .  .  .  .  .  .  .  .  .  .  .  .  A  .  .  .  .  .  .  .  .  .  .  .  .  .  .  . | T  .  .  .  .  .  .  .  .  .  .  .  .  .  .  .  .  .  .  .  .  .  .  .  .  .  G  G  G  G  G  .  . | C  .  .  .  .  .  .  .  .  .  .  .  .  .  .  .  .  .  .  .  .  .  .  .  .  .  T  T  T  T  T  T  T | C  .  .  .  .  .  T  .  .  .  .  .  .  .  .  .  .  .  .  .  .  .  .  .  .  .  .  .  .  .  .  .  . | T  .  .  .  .  .  .  .  .  .  .  .  .  .  .  .  .  .  .  .  .  .  .  .  .  .  .  .  .  .  .  G  G | A  .  .  .  .  .  .  .  .  .  .  .  .  .  .  .  .  .  .  .  .  .  .  .  .  .  .  .  .  .  .  .  G | C  .  .  .  .  .  .  .  .  .  .  .  .  .  .  .  .  .  .  .  .  .  .  .  .  .  .  .  .  .  .  T  T | G  .  .  .  .  .  .  .  .  .  .  .  .  .  .  .  .  .  .  .  .  .  .  .  .  .  A  A  A  A  A  .  . | G  .  .  .  .  .  .  .  .  .  .  .  .  .  .  .  .  .  .  .  .  .  .  .  .  .  .  .  .  .  .  .  . | A  .  .  .  .  .  .  G  .  .  .  .  .  .  .  .  .  .  .  .  .  .  .  .  .  .  .  .  .  .  .  .  . | A  .  .  .  .  .  .  .  .  .  G  .  .  .  .  .  .  .  .  .  .  G  .  .  .  .  .  .  .  .  .  .  . | T  .  .  .  .  .  .  .  .  .  .  .  .  .  .  .  .  .  .  .  .  .  .  .  .  .  .  .  .  .  .  C  . | T  .  .  .  .  .  .  .  .  .  .  .  .  .  .  .  .  .  .  .  .  .  .  .  .  .  .  .  .  .  .  G  G | G  .  .  .  A  A  A  A  .  A  .  .  .  .  .  .  .  .  .  .  .  .  .  .  .  .  A  A  A  A  A  A  A | T  .  .  .  .  .  .  .  .  .  .  .  .  .  .  .  .  .  .  .  .  .  .  .  .  .  .  .  .  .  .  .  A | C  .  .  .  .  .  .  .  .  .  T  .  .  .  .  .  .  .  .  .  .  T  .  .  .  .  .  .  .  .  .  .  . | G  .  .  .  .  .  .  .  .  .  .  .  .  .  .  .  .  .  .  .  .  .  .  .  .  .  .  .  .  .  .  A  . | C  .  .  .  .  .  .  .  .  .  .  .  .  .  .  .  .  .  .  .  T  .  .  .  .  .  .  .  .  .  .  .  . | A  .  .  .  .  .  .  .  .  .  .  .  .  .  .  .  .  .  .  .  .  .  .  .  .  .  .  .  .  .  .  G  G |

Supplementary Table 3 Segregating sites between the concatenated sequences of the *cox1* gene of the haplotypes of *E. multilocularis* identified in this study (haplotypes A11 to A26) compared with the sequence of the already described haplotypes of *E. multilocularis* by Nakao et al, 2009 (haplotypes A1-A10 excluding O1). Nucleotide positions are numbered from the first nucleotide of the gene.
