# Supplementary material for: Genetic diversity of Echinococcus multilocularis and Echinococcus granulosus sensu lato in Kyrgyzstan: The A2 haplotype of E. multilocularis is the predominant variant infecting humans
Source: PLoS Negl Trop Dis. 2020 May 13;14(5):e0008242. doi: 10.1371/journal.pntd.0008242 (PMC7219741; doi:10.1371/journal.pntd.0008242)
Supplement: S4 Table — (DOCX) [file pntd.0008242.s004.docx]

| Nucleotide position | | | | | | | | | | | | | | | | | | | | | | | | | | |
| --- | --- | --- | --- | --- | --- | --- | --- | --- | --- | --- | --- | --- | --- | --- | --- | --- | --- | --- | --- | --- | --- | --- | --- | --- | --- | --- |
|  | 18 | 31 | 31 | 42 | 51 | 106 | 183 | 189 | 270 | 325 | 349 | 368 | 505 | 512 | 542 | 576 | 585 | 597 | 620 | 633 | 641 | 669 | 717 | 723 | 750 | 765 |
| Eg01  Eg03  Eg32  Eg33  EgCL04  EgKyr1  EgKyr2  EgKyr3  EgKyr4  EgKyr5  EgKyr6  EgKyr7  EgKyr8  EgKyr9  EgKyr10  EgKyr11  EgKyr12  EgKyr13  EgKyr14  EgKyr15  EgKyr16  EgKyr17  EgKyr18  EgKyr19 | .  .  .  .  .  .  .  .  .  .  .  .  .  .  .  .  .  .  .  G  .  .  .  . | A  .  .  .  .  .  .  .  .  .  .  .  .  G  .  .  .  .  .  .  .  .  .  . | T  .  .  C  .  .  .  .  .  .  .  .  .  .  .  .  .  .  .  .  .  .  .  . | T  .  .  C  .  .  .  .  .  .  .  .  .  .  .  .  .  .  .  .  .  .  .  . | C  .  .  .  .  .  .  .  .  .  .  .  T  .  .  .  G  .  .  .  .  .  .  . | T  .  .  .  .  .  .  .  .  .  .  .  .  .  .  .  .  .  .  .  .  C  .  . | T  .  .  .  .  .  .  .  .  .  C  .  .  C  .  .  .  .  .  .  .  C  .  . | C  .  .  .  .  .  .  .  .  .  .  .  .  .  .  .  .  .  .  .  T  .  .  . | T  .  .  .  .  .  .  .  .  .  C  .  .  .  .  .  .  .  .  .  .  .  .  . | A  .  .  .  .  .  .  .  .  G  .  .  .  .  .  .  .  .  .  .  .  .  .  . | G  .  .  .  .  .  .  .  .  .  .  .  .  .  .  .  .  .  .  .  C  .  .  . | C.  .  .  .  .  .  .  .  .  .  .  .  .  .  .  .  .  .  T  .  .  .  .  . | C  T  .  .  .  .  .  .  .  .  .  .  .  .  .  .  .  .  .  .  .  .  .  . | G  .  .  .  .  .  .  .  .  .  .  .  .  .  .  .  .  T  .  .  .  .  .  . | C  .  .  .  .  .  .  .  T  .  .  .  .  .  .  .  .  .  .  .  .  .  .  . | T  .  .  .  .  .  .  C  .  .  .  .  .  .  .  .  .  .  .  .  .  .  .  . | A  .  .  .  .  .  .  .  .  .  .  .  .  .  .  .  .  .  .  G  .  .  .  . | G  .  .  .  .  .  .  .  .  .  .  .  .  .  .  .  .  .  .  .  .  .  A  . | C  .  .  .  .  .  .  .  .  .  T  .  .  .  .  .  .  .  .  .  .  .  .  . | C  .  .  .  .  .  .  .  .  T  .  .  .  .  .  .  .  .  .  .  .  .  .  . | A  .  .  .  .  .  .  .  .  .  .  .  .  .  .  .  G  .  .  .  .  .  .  . | A  .  .  .  .  .  G  .  .  .  .  G  .  .  .  .  .  .  .  .  .  .  .  . | C  .  T  .  .  .  .  .  .  .  .  .  .  .  .  .  .  .  .  .  .  .  .  . | T  .  .  .  .  .  .  .  .  .  .  .  .  .  .  C  .  .  .  .  .  .  .  . | A  .  .  .  .  .  .  .  .  .  .  .  .  .  .  .  .  G  .  .  .  .  .  . | T  .  .  .  .  .  .  .  .  .  .  .  .  C  .  .  .  .  .  .  .  C  .  . |

Supplementary Table 4 Segregating sites between the concatenated sequences of the cox1 gene of the haplotypes of *E. granulosus sensu stricto* identified in this study (haplotypes EgKyr1-EgKyr19) compared with the sequence of the already described haplotypes of *E. granulosus s.s.* Eg01 (JQ250806), Eg03 (JQ250808), Eg32 (AB688609), Eg33 (AB688610) and EgCl04 (KX227119). Nucleotide positions are numbered from the first nucleotide of the gene.

| Nucleotide position | | | | | | | | | | | | | | | |
| --- | --- | --- | --- | --- | --- | --- | --- | --- | --- | --- | --- | --- | --- | --- | --- |
|  | 800 | 810 | 918 | 972 | 990 | 1003 | 1056 | 1143 | 1239 | 1326 | 1422 | 1497 | 1511 | 1536 | 1592 |
| Eg01  Eg03  Eg32  Eg33  EgCL04  EgKyr1  EgKyr2  EgKyr3  EgKyr4  EgKyr5  EgKyr6  EgKyr7  EgKyr8  EgKyr9  EgKyr10  EgKyr11  EgKyr12  EgKyr13  EgKyr14  EgKyr15  EgKyr16  EgKyr17  EgKyr18  EgKyr19 | C  .  .  .  .  .  .  .  .  .  .  .  .  .  .  .  .  .  .  .  .  .  .  T | C  .  .  .  .  .  .  .  .  .  .  .  T  .  .  .  .  .  T  .  .  .  .  . | C  .  .  .  .  .  .  .  T  .  .  .  .  .  .  T  .  .  .  .  .  .  .  . | A  .  .  .  .  .  .  .  .  .  .  .  .  .  G  .  .  .  G  .  .  .  .  . | G  .  .  .  .  A  .  .  .  .  .  .  .  .  .  .  .  .  .  .  .  .  .  . | A  .  .  .  .  G  .  .  .  .  .  .  .  .  .  .  .  .  .  .  .  .  .  . | G  .  .  .  .  .  .  .  .  .  .  .  .  .  .  .  .  .  .  .  A  .  .  . | T  .  .  .  C  .  .  .  .  .  .  .  .  .  .  .  .  .  .  .  .  .  .  . | T  .  .  .  .  .  .  .  .  .  C  .  .  C  .  .  .  .  .  .  .  C  .  . | C  .  .  .  .  .  .  .  .  .  .  .  .  .  .  .  .  .  .  .  T  .  .  . | G  .  .  .  .  .  .  .  .  .  .  .  .  .  .  .  .  .  .  .  .  .  .  T | T  .  .  .  .  .  .  .  .  .  .  C  .  .  .  .  .  .  .  .  .  .  .  . | A  .  .  .  .  .  .  .  .  .  .  .  .  .  G  .  .  .  .  .  .  .  .  . | C  .  .  .  .  .  .  .  .  .  .  .  .  .  .  .  .  .  T  .  .  .  .  . | C  .  .  .  .  .  .  .  .  .  .  .  .  .  .  .  .  .  T  .  .  .  .  . |

Supplementary Table 4 (continuation) Segregating sites between the concatenated sequences of the cox1 gene of the haplotypes of *E. granulosus sensu stricto* identified in this study (haplotypes EgKyr1-EgKyr19) compared with the sequence of the already described haplotypes of *E. granulosus s.s.* Eg01 (JQ250806), Eg03 (JQ250808), Eg32 (AB688609), Eg33 (AB688610) and EgCl04 (KX227119). Nucleotide positions are numbered from the first nucleotide of the gene.
